# *N*-Isopropyl­benzamide

**DOI:** 10.1107/S1600536808012804

**Published:** 2008-05-07

**Authors:** Erik M. van Oosten, Alan J. Lough, Neil Vasdev

**Affiliations:** a Department of Chemistry, University of Toronto, 80 St George Street, Toronto, Ontario, Canada M5S 3H6; b PET Centre, Centre for Addiction and Mental Health, and Department of Psychiatry, University of Toronto, 250 College Street, Toronto, Ontario, Canada M5T 1R8

## Abstract

In the title compound, C_10_H_13_NO, the dihedral angle between the amide group and the phenyl ring is 30.0 (3)°. In the crystal structure, inter­molecular N—H⋯O hydrogen bonds link mol­ecules into one-dimensional chains along the *a* axis.

## Related literature

For related literature, see: Clayden *et al.* (2006 ▶); Kopka *et al.* (2005 ▶); Smart (2001 ▶); Van Waarde *et al.* (2004 ▶); Stephenson, Wilson *et al.* (2008 ▶); Stephenson, van Oosten *et al.* (2008 ▶).
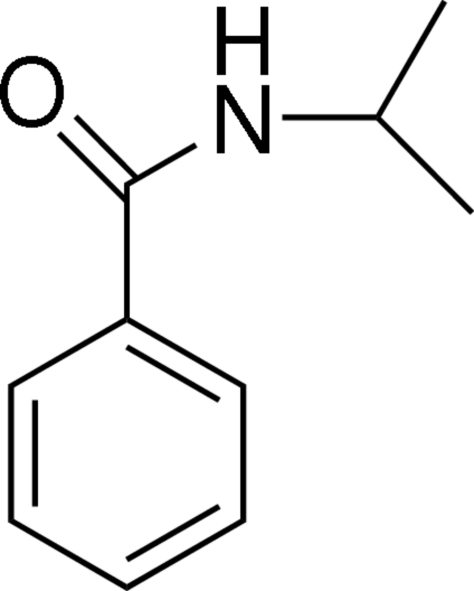


## Experimental

### 

#### Crystal data

C_10_H_13_NO
*M* *_r_* = 163.21
Monoclinic, 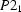

*a* = 5.0093 (7) Å
*b* = 10.1250 (13) Å
*c* = 9.6714 (14) Å
β = 104.133 (7)°
*V* = 475.68 (11) Å^3^
*Z* = 2
Mo *K*α radiation
μ = 0.07 mm^−1^
*T* = 150 (1) K
0.14 × 0.13 × 0.08 mm

#### Data collection

Bruker Nonius KappaCCD diffractometer
Absorption correction: multi-scan (*SORTAV*; Blessing 1995 ▶) *T*_min_ = 0.954, *T*_max_ = 0.996
2462 measured reflections
887 independent reflections
621 reflections with *I* > 2σ(*I*)
*R*_int_ = 0.061

#### Refinement

*R*[*F* ^2^ > 2σ(*F* ^2^)] = 0.057
*wR*(*F* ^2^) = 0.148
*S* = 1.06
887 reflections
114 parameters
1 restraint
H atoms treated by a mixture of independent and constrained refinement
Δρ_max_ = 0.18 e Å^−3^
Δρ_min_ = −0.21 e Å^−3^

### 

Data collection: *COLLECT* (Nonius, 2002 ▶); cell refinement: *DENZO-SMN* (Otwinowski & Minor, 1997 ▶); data reduction: *DENZO-SMN*; program(s) used to solve structure: *SIR92* (Altomare *et al.*, 1994 ▶); program(s) used to refine structure: *SHELXTL* (Sheldrick, 2008 ▶); molecular graphics: *PLATON* (Spek, 2003 ▶); software used to prepare material for publication: *SHELXTL*.

## Supplementary Material

Crystal structure: contains datablocks global, I. DOI: 10.1107/S1600536808012804/hb2729sup1.cif

Structure factors: contains datablocks I. DOI: 10.1107/S1600536808012804/hb2729Isup2.hkl

Additional supplementary materials:  crystallographic information; 3D view; checkCIF report

## Figures and Tables

**Table 1:** Hydrogen-bond geometry (Å, °)

*D*—H⋯*A*	*D*—H	H⋯*A*	*D*⋯*A*	*D*—H⋯*A*
N1—H1N⋯O1^i^	0.83 (5)	2.10 (5)	2.890 (5)	160 (5)

Symmetry code: (i) 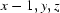.

**Fig. 1.**
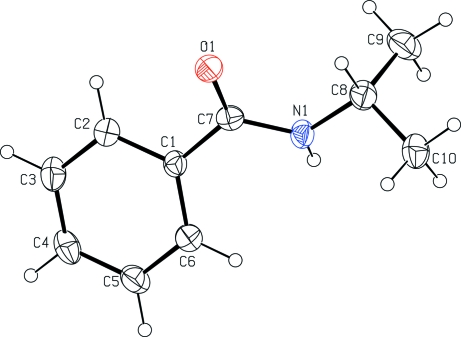
The molecular structure of (I) showing 30% probability displacement ellipsoids (arbitrary spheres for H atoms).

**Fig. 2.**
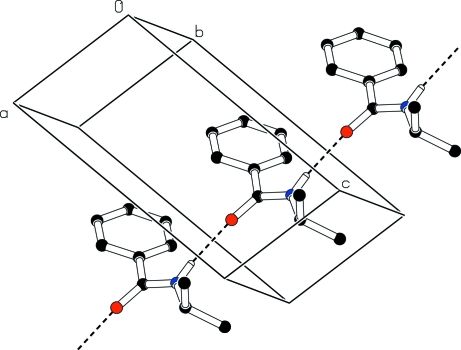
Part of the crystal structure of (I) showing hydrogen bonds as dashed lines.

## supplementary crystallographic information

### Comment

The isopropylamine moiety is a common structural feature in many pharmaceutical compounds, in particular among β-adrenergic receptor antagonists (β-blockers) (Van Waarde *et al.*, 2004; Kopka *et al.*, 2005). Recent work in our laboratory (Stephenson, Wilson *et al.*, 2008; Stephenson, van Oosten *et al.*, 2008) and others (Van Waarde *et al.*, 2004; Kopka *et al.*, 2005) has focused on developing β-blockers labeled with the positron emitting isotope fluorine-18 (t_1/2_ = 109.7 min) at the isopropyl moiety for medical imaging with positron emission tomography. It is established that substitution of fluorine into a drug often enhances its biological properties (Smart, 2001). Our goal is to structurally characterize the isopropylamine group for comparison with fluorinated analogs developed in our laboratory. Herein we report the single-crystal X-ray structure of the title compound, (I), (Fig. 1).

The dihedral angle between the essentially planar set of atoms C7/O1/N1/C8 [r.m.s. deviation 0.006 Å] and the benzene ring (C1–C6) in (I) is 30.0 (3)°. In the crystal structure, intermolecular N—H···O hydrogen bonds link molecules into one-dimensional chains along the *a* axis (Table 1, Fig. 2).

### Experimental

*N*-Isopropylbenzamide was made according to a literature procedure (Clayden *et al.*, 2006), with minor modifications. Benzoyl chloride (0.825 ml, 7.11 mmol) was added to CH_2_Cl_2_ (17 ml, 0.4 *M*) under nitrogen. The mixture was cooled in an ice bath to 273 K and stirred for 10 min. Isopropylamine (1.8 ml, 21.33 mmol) was added dropwise. Upon completion of this addition the ice bath was removed and the reaction mixture was stirred at room temperature for 1.5 h. When the starting material was consumed (monitored by TLC) the reaction mixture was diluted with H_2_O (150 ml), extracted with CH_2_Cl_2_ (3 × 50 ml), washed with H_2_O (2 × 100 ml) followed by brine (2 × 100 ml), dried over Na_2_SO_4_, and concentrated. No further purification was necessary. Colourless blocks of (I) were obtained by slow evaporation of a solution of the title compound in CDCl_3_. ^1^H NMR (CDCl_3_, 300 MHz) d = 7.78–7.67 (m, 2H), 7.51–7.36 (m, 3H), 5.99 (br, 1H), 4.37–4.18 (m, 1H), 1.25 (d, J = 6.5 Hz, 6H) ^13^C NMR (CDCl_3_, 75 MHz) d = 166.9, 135.2, 131.5, 128.7, 127.0, 42.1, 23.1.

### Refinement

In the absence of significant anamlous dispersion effects, Friedel pairs were merged before refinement. The H atoms bonded to C atoms were placed in calculated positions with C—H = 0.95 Å and 0.98 Å (methyl). They were included in the refinement in the riding-model approximation with *U*_iso_(H) = 1.2*U*_eq_(C) or *U*_iso_(H) = 1.5*U*_eq_(C) for methyl H atoms. The position of the H atom bonded to the N atom was refined independently with *U*_iso_(H) = 1.2*U*_eq_(N).

### Crystal data

**Table:**

C_10_H_13_NO	*F*_000_ = 176
*M**_r_* = 163.21	*D*_x_ = 1.140 Mg m^−^^3^
Monoclinic, *P*2_1_	Mo *K*α radiation λ = 0.71073 Å
Hall symbol: P 2yb	Cell parameters from 2462 reflections
*a* = 5.0093 (7) Å	θ = 3.0–25.0º
*b* = 10.1250 (13) Å	µ = 0.07 mm^−^^1^
*c* = 9.6714 (14) Å	*T* = 150 (1) K
β = 104.133 (7)º	Block, colourless
*V* = 475.68 (11) Å^3^	0.14 × 0.13 × 0.08 mm
*Z* = 2	

### Data collection

**Table:**

Bruker Nonius KappaCCD diffractometer	887 independent reflections
Radiation source: fine-focus sealed tube	621 reflections with *I* > 2σ(*I*)
Monochromator: graphite	*R*_int_ = 0.061
Detector resolution: 9 pixels mm^-1^	θ_max_ = 25.0º
*T* = 150(1) K	θ_min_ = 3.0º
φ scans and ω scans with κ offsets	*h* = −5→5
Absorption correction: multi-scan(SORTAV; Blessing 1995)	*k* = −12→10
*T*_min_ = 0.954, *T*_max_ = 0.996	*l* = −11→11
2462 measured reflections	

### Refinement

**Table:**

Refinement on *F*^2^	Secondary atom site location: difference Fourier map
Least-squares matrix: full	Hydrogen site location: inferred from neighbouring sites
*R*[*F*^2^ > 2σ(*F*^2^)] = 0.057	H atoms treated by a mixture of independent and constrained refinement
*wR*(*F*^2^) = 0.148	*w* = 1/[σ^2^(*F*_o_^2^) + (0.0784*P*)^2^] where *P* = (*F*_o_^2^ + 2*F*_c_^2^)/3
*S* = 1.06	(Δ/σ)_max_ < 0.001
887 reflections	Δρ_max_ = 0.18 e Å^−^^3^
114 parameters	Δρ_min_ = −0.21 e Å^−^^3^
1 restraint	Extinction correction: none
Primary atom site location: structure-invariant direct methods	

### Special details

**Table:**

Geometry. All e.s.d.'s (except the e.s.d. in the dihedral angle between two l.s. planes) are estimated using the full covariance matrix. The cell e.s.d.'s are taken into account individually in the estimation of e.s.d.'s in distances, angles and torsion angles; correlations between e.s.d.'s in cell parameters are only used when they are defined by crystal symmetry. An approximate (isotropic) treatment of cell e.s.d.'s is used for estimating e.s.d.'s involving l.s. planes.
Refinement. Refinement of *F*^2^ against ALL reflections. The weighted *R*-factor *wR* and goodness of fit *S* are based on *F*^2^, conventional *R*-factors *R* are based on *F*, with *F* set to zero for negative *F*^2^. The threshold expression of *F*^2^ > σ(*F*^2^) is used only for calculating *R*-factors(gt) *etc*. and is not relevant to the choice of reflections for refinement. *R*-factors based on *F*^2^ are statistically about twice as large as those based on *F*, and *R*-factors based on ALL data will be even larger.

### Fractional atomic coordinates and isotropic or equivalent isotropic displacement parameters (Å^2^)

**Table:**

	*x*	*y*	*z*	*U*_iso_*/*U*_eq_	
O1	0.6751 (6)	0.1703 (3)	0.8045 (3)	0.0424 (8)	
N1	0.2696 (8)	0.1717 (4)	0.8673 (4)	0.0411 (10)	
H1N	0.103 (11)	0.188 (5)	0.839 (5)	0.049*	
C1	0.3226 (9)	0.3127 (4)	0.6749 (5)	0.0350 (11)	
C2	0.4247 (10)	0.3165 (5)	0.5544 (5)	0.0452 (13)	
H2A	0.5601	0.2545	0.5432	0.054*	
C3	0.3286 (11)	0.4116 (6)	0.4493 (5)	0.0535 (14)	
H3A	0.3957	0.4126	0.3655	0.064*	
C4	0.1377 (10)	0.5034 (5)	0.4666 (6)	0.0516 (14)	
H4A	0.0734	0.5682	0.3951	0.062*	
C5	0.0391 (11)	0.5014 (5)	0.5883 (6)	0.0523 (14)	
H5A	−0.0912	0.5656	0.6010	0.063*	
C6	0.1296 (9)	0.4064 (5)	0.6915 (5)	0.0451 (13)	
H6A	0.0597	0.4051	0.7744	0.054*	
C7	0.4356 (8)	0.2117 (4)	0.7864 (4)	0.0362 (12)	
C8	0.3461 (10)	0.0743 (5)	0.9808 (5)	0.0474 (13)	
H8A	0.5489	0.0586	0.9996	0.057*	
C9	0.1984 (13)	−0.0561 (5)	0.9351 (6)	0.0648 (16)	
H9A	0.2525	−0.0903	0.8512	0.097*	
H9B	−0.0011	−0.0417	0.9116	0.097*	
H9C	0.2488	−0.1200	1.0132	0.097*	
C10	0.2833 (11)	0.1271 (6)	1.1160 (5)	0.0556 (15)	
H10A	0.3743	0.2126	1.1400	0.083*	
H10B	0.3510	0.0647	1.1942	0.083*	
H10C	0.0839	0.1380	1.1013	0.083*	

### Atomic displacement parameters (Å^2^)

**Table:**

	*U*^11^	*U*^22^	*U*^33^	*U*^12^	*U*^13^	*U*^23^
O1	0.0319 (17)	0.0442 (18)	0.0515 (17)	0.0024 (15)	0.0112 (13)	0.0032 (16)
N1	0.0326 (19)	0.046 (2)	0.047 (2)	0.003 (2)	0.0149 (18)	0.012 (2)
C1	0.033 (2)	0.035 (2)	0.039 (3)	−0.006 (2)	0.012 (2)	0.000 (2)
C2	0.049 (3)	0.044 (3)	0.046 (3)	0.001 (2)	0.019 (2)	0.003 (2)
C3	0.057 (3)	0.061 (3)	0.046 (3)	−0.003 (3)	0.019 (3)	0.012 (3)
C4	0.049 (3)	0.051 (3)	0.050 (3)	−0.002 (3)	0.004 (3)	0.020 (3)
C5	0.053 (3)	0.040 (3)	0.066 (3)	0.007 (3)	0.017 (3)	0.013 (3)
C6	0.046 (3)	0.042 (3)	0.051 (3)	0.003 (2)	0.017 (2)	0.005 (3)
C7	0.035 (3)	0.034 (3)	0.037 (2)	−0.004 (2)	0.005 (2)	−0.008 (2)
C8	0.041 (3)	0.054 (3)	0.048 (3)	0.012 (3)	0.012 (2)	0.017 (3)
C9	0.085 (4)	0.043 (3)	0.064 (3)	0.010 (3)	0.014 (3)	0.012 (3)
C10	0.062 (3)	0.062 (4)	0.044 (3)	−0.001 (3)	0.015 (2)	0.012 (3)

### Geometric parameters (Å, °)

**Table:**

O1—C7	1.242 (5)	C5—C6	1.380 (7)
N1—C7	1.337 (6)	C5—H5A	0.9500
N1—C8	1.455 (6)	C6—H6A	0.9500
N1—H1N	0.83 (5)	C8—C10	1.516 (7)
C1—C2	1.383 (6)	C8—C9	1.525 (8)
C1—C6	1.392 (6)	C8—H8A	1.0000
C1—C7	1.493 (6)	C9—H9A	0.9800
C2—C3	1.398 (8)	C9—H9B	0.9800
C2—H2A	0.9500	C9—H9C	0.9800
C3—C4	1.373 (7)	C10—H10A	0.9800
C3—H3A	0.9500	C10—H10B	0.9800
C4—C5	1.384 (7)	C10—H10C	0.9800
C4—H4A	0.9500		
			
C7—N1—C8	124.0 (4)	O1—C7—N1	122.2 (4)
C7—N1—H1N	118 (4)	O1—C7—C1	121.0 (4)
C8—N1—H1N	117 (4)	N1—C7—C1	116.7 (4)
C2—C1—C6	119.2 (4)	N1—C8—C10	109.9 (4)
C2—C1—C7	118.3 (4)	N1—C8—C9	110.4 (4)
C6—C1—C7	122.4 (4)	C10—C8—C9	111.5 (4)
C1—C2—C3	120.0 (5)	N1—C8—H8A	108.3
C1—C2—H2A	120.0	C10—C8—H8A	108.3
C3—C2—H2A	120.0	C9—C8—H8A	108.3
C4—C3—C2	120.3 (5)	C8—C9—H9A	109.5
C4—C3—H3A	119.9	C8—C9—H9B	109.5
C2—C3—H3A	119.9	H9A—C9—H9B	109.5
C3—C4—C5	119.8 (5)	C8—C9—H9C	109.5
C3—C4—H4A	120.1	H9A—C9—H9C	109.5
C5—C4—H4A	120.1	H9B—C9—H9C	109.5
C6—C5—C4	120.2 (5)	C8—C10—H10A	109.5
C6—C5—H5A	119.9	C8—C10—H10B	109.5
C4—C5—H5A	119.9	H10A—C10—H10B	109.5
C5—C6—C1	120.5 (4)	C8—C10—H10C	109.5
C5—C6—H6A	119.8	H10A—C10—H10C	109.5
C1—C6—H6A	119.8	H10B—C10—H10C	109.5

### Hydrogen-bond geometry (Å, °)

**Table:**

*D*—H···*A*	*D*—H	H···*A*	*D*···*A*	*D*—H···*A*
N1—H1N···O1^i^	0.83 (5)	2.10 (5)	2.890 (5)	160 (5)

Symmetry codes: (i) *x*−1, *y*, *z*.
